# The Optimization of a Microfluidic CTC Filtering Chip by Simulation

**DOI:** 10.3390/mi8030079

**Published:** 2017-03-04

**Authors:** Huan Li, Jianfeng Chen, Wenqiang Du, Youjun Xia, Depei Wang, Gang Zhao, Jiaru Chu

**Affiliations:** 1 Department of Precision Machinery and Precision Instrumentation, University of Science and Technology of China, Hefei 230031, China; lh1991@mail.ustc.edu.cn (H.L.); cjfowen@mail.ustc.edu.cn (J.C.); bendu@mail.ustc.edu.cn (W.D.); youjun@mail.ustc.edu.cn (Y.X.); depeiw@mail.ustc.edu.cn (D.W.); jrchu@ustc.edu.cn (J.C.); 2 Micro&Nano Engineering Laboratory, University of Science and Technology of China, Hefei 230031, China

**Keywords:** circulating tumor cells (CTCs), circulating tumor cell, microfilter, volume of fluid (VOF), volume of fluid, simulation, area strain, cellular damage

## Abstract

The detection and separation of circulating tumor cells (CTCs) are crucial in early cancer diagnosis and cancer prognosis. Filtration through a thin film is one of the size and deformability based separation methods, which can isolate rare CTCs from the peripheral blood of cancer patients regardless of their heterogeneity. In this paper, volume of fluid (VOF) multiphase flow models are employed to clarify the cells’ filtering processes. The cells may deform significantly when they enter a channel constriction, which will induce cell membrane stress and damage if the area strain is larger than the critical value. Therefore, the cellular damage criterion characterized by membrane area strain is presented in our model, i.e., the lysis limit of the lipid bilayer is taken as the critical area strain. Under this criterion, we discover that the microfilters with slit-shaped pores do less damage to cells than those with circular pores. The influence of contact angle between the microfilters and blood cells on cellular injury is also discussed. Moreover, the optimal film thickness and flux in our simulations are obtained as 0.5 μm and 0.375 mm/s, respectively. These findings will provide constructive guidance for the improvement of next generation microfilters with higher throughput and less cellular damage.

## 1. Introduction

Circulating tumor cells (CTCs) are cancerous cells in the peripheral blood shed from primary tumors. They are carried around in the circulation of a cancer patient prior to the emergence of clinical symptoms, which means that CTCs can be a harbinger of cancer formation and metastasis [1,2]. Therefore, CTC detection and separation are vital in early cancer diagnosis, cancer prognosis, and therapeutic assessment [3,4,5]. However, CTCs in cancer patients are very rare, about 1 per milliliter. The total amount of blood in the human body is about 5000 mL. Even if all of the blood in the human body is filtered at once, the total number that we can obtain is 5000 or so. Therefore, to illuminate the role that CTCs play in the development of cancer, effective strategies for separating extremely low concentrations of tumor cells must be put forward. Up to now, the methodology of the separation of CTCs can be divided into two major categories: biophysical methods and biochemical methods [6,7,8,9,10]. The biochemical methods mainly utilize tumor-specific antibodies, usually epithelial cell adhesion molecule (EpCAM), to identify CTCs, and thus they are limited by the fact that the expression of certain biomarkers is a highly heterogeneous and unstable process [11]. Moreover, it is hard to select suitable antibodies before we know which type of CTCs to screen for.

To work around these issues, various label free methods have been developed utilizing the cells biophysical properties such as size [12], deformability [13], inertia [14,15,16], dielectric attributes [17], optical properties [18], and acoustic features [19]. Among these biophysical approaches, the filtration strategy based on the fact that CTCs are typically stiffer and larger than normal blood cells [20,21] is prevailing for its ease of operation, structural simplicity, high throughput, and low cost. The microfilters based on size and deformability can isolate most of the CTCs, whereas the conventional biomedical methods can only isolate a small subset whose biomarker is precisely specific to the antibody, taking the genetic instability and heterogeneity of the CTCs into account. Recently, many micro-filtering systems have been explored [5,22,23]. However, the mechanism and optimal parameters of filtration are still unclear due to the difficulty in observing the dynamic filtering process. In this regard, it is better to understand the filtering processes and optimize the microfilters by simulation.

In the previous simulations, the filtering process is described as a mechanical issue of interaction among the cell, wall, and fluid. The cell can be modeled as a deformable solid or liquid droplet [24,25,26,27,28,29,30]. Considering the scale of the filtering process, the internal structures of the cell, such as the cell nucleus and cytoskeleton, can be ignored if we do not care about the subcellular-level response. Furthermore, compared to the solid model, the liquid model has obvious advantages in computation convergence under large deformation conditions [25,27,31]. In this paper, volume of fluid (VOF) multiphase flow models [26,27,29,30,32] are adopted for determining the cells’ filtering processes, which can provide insights into the design of CTC microfilters. In the filtering process, CTCs should be trapped while normal blood cells should not be. Moreover, the cellular injuries should be as low as possible for the benefit of the subsequent research. Although some simulation results for CTC microfilters have been reported before, most of them focused on the influence of filtering channel geometries on the pressure drop [25,27,29,32] and entrance time [25]. To the best of our knowledge, the impact of the filtering process on cellular injuries is not well elucidated and the cellular damage characterized by membrane area strain remains elusive.

In general, the main cellular damage in the physical process is caused by the excessive tensile stress of the cell membrane [33,34,35]. As reported in the previous literature, the 5%–6% cell area strain will lead to lysis of the lipid bilayer under a dynamic situation with low strain rates, which denotes that the area strain can be an indicator of cellular injury [36,37,38]. Thus, the average lysis limit of 5.5% is taken as a critical area strain in our model. The cells are considered damaged if the area strain is larger than the critical value. Under the guidance of this criterion, we explored the effect of different parameters, such as filter geometric pattern, filtering film thickness, and flow rate, on cell membrane strain in dynamic processes. A better design pattern that is suitable for the microfilters is determined as the slit-shaped one and the optimized thickness and flow rate are obtained as well. That is, with these optimal parameter settings, minimal damage is done to the filtered cells. These findings will direct the fabrication of next generation microfilters that meet the higher throughput, lower cellular injury requirements.

## 2. Methods

**Figure 1:**
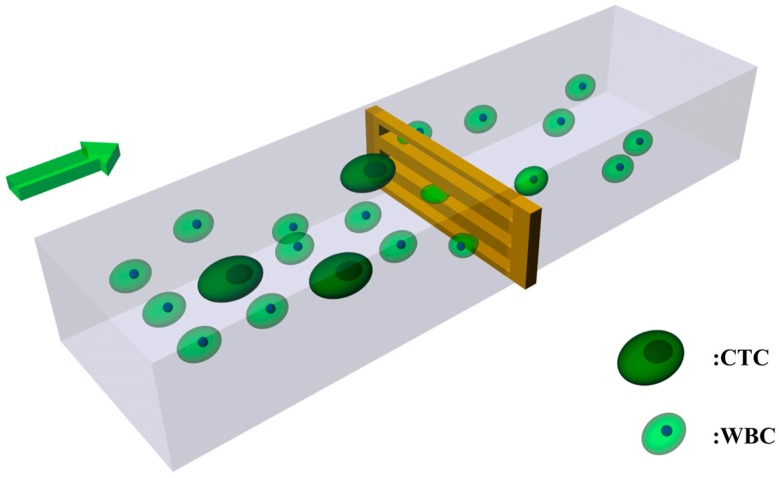
Schematic diagram of the filtering process.

The commercial software ANSYS Fluent is used to perform the transient simulation in this paper, and the VOF models are built to explore the behavior of cells when they traverse the filtering channels. In the model, cells are regarded as liquid droplets confined by the surface tension and contact angle. The contact angle depicts the adhesion between the cell and microfilter film. The surface tension represents the restraint of the lipid bilayer to the cell. Typically, CTCs have larger surface tension than normal blood cells. Note that red blood cells are ignored in the present study since they are softer and smaller than white blood cells (WBCs) and the traversing study of normal blood cells can be replaced by that of WBCs. To reach the purpose of enrichment of CTCs, the filtering film should catch all the CTCs flowing by, while allowing most of WBCs to pass through. The filtering process is shown in Figure 1.

In the cases reported here, two-equation turbulence models named realizable *k*-ε models are used for numerical accuracy, in which the turbulence kinetic energy, *k*, and its rate of dissipation, ε, are introduced. The pressure-implicit with splitting of operators (PISO) scheme that is suitable for transient computation is employed for the pressure-velocity coupling. Meanwhile, the pressure staggering option (PRESTO) method is applied for pressure and the Geo-Reconstruct method is used for volume fraction spatial discretization available in Fluent. For the boundary settings, the inlet of the channel is set as the constant flow rate and the outlet as the pressure outlet. The periodic boundary condition is set at the lateral face of the fluid body while no-slip and stationary conditions are set at the channel walls. The parameters set in the VOF models are shown in Table 1. There are two phases in the present research, the main phase blood and secondary phase cells. The diameter of normal blood cells is set as 16 μm, since only a small amount of WBCs are larger than that. On the contrary, the diameter of CTCs is set as 10 μm, since the majority of CTCs are larger than that. The contact angle in the models characterizes the affinity between the cells and the filtering film. A 180° contact angle means that cells are not adhesive to the filtering film at all, while a 90° contact angle means that the cells have the same affinity for the filtering film as the blood. As is known to us, different cells have different surface tensions, and the typical values of cell properties in the previous literature are used in our model.

**Figure 2:**
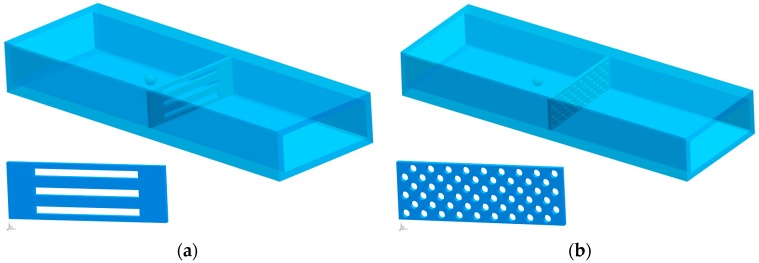
The periodic boundary condition is set at the lateral side of the fluid body: (**a**) filtering film with 100 μm × 7 μm slit-shaped pores; (**b**) filtering film with ϕ 7 μm circular pores. The section of the fluid body is 150 μm × 41 μm, and the total length is 500 μm.

At present, a variety of types of filtering films have been investigated, among which the microfilters with circular pores and rectangular pores have been mostly used. For a meaningful contrast of micro-filtering films composed of the array of circular and slit-shaped pores, we fixed the porosity and characteristic dimension of the microfilters at a constant value. The patterns of filtering films to be compared are illustrated in Figure 2. The diameter of the circles and the width of the rectangles are set as 7 μm and these two models have equivalent pore areas. Although the models established in our simulations are finite, the periodic boundary condition set at the lateral side of the fluid body means that the area of the filtering films is considered infinite.

## 3. Results and Discussion

The separation of CTCs from normal blood cells can be achieved by arresting CTCs while letting WBCs through. In this study, the surface tension is a marker to discriminate the blood cells. Blood cells with different surface tensions reach different deformations and shapes, even if their volumes are identical. Due to the greater surface tension of CTCs, they are tougher than WBCs to pass through smaller constrictions. We characterize the damage of blood cells by the membrane area strain of the passed WBCs and the captured CTCs. In this section, a better pattern of the filtering film is obtained, after which a parametric study is performed to investigate the effects of film thickness, contact angle, and inlet velocity on the CTCs’ separation process.

### 3.1. Geometry Comparison Analysis

#### 3.1.1. Cellular Damage of the Passed WBCs

**Figure 3:**
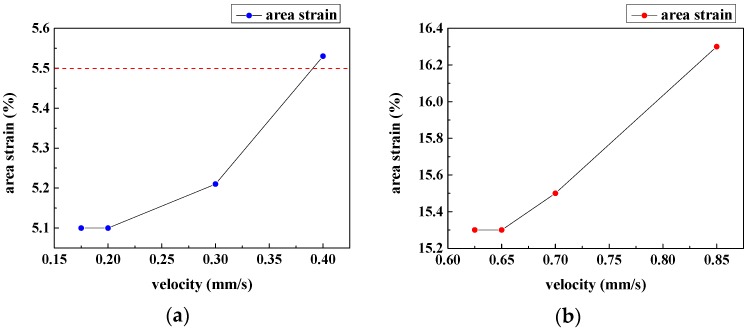
Membrane area strain of white blood cells (WBCs) at various inlet velocities: (**a**) area strain of WBCs passing through the microfilter with slit-shaped pores; (**b**) area strain of WBCs passing through the microfilter with circular pores.

A number of simulations of the processes of blood cells transiting through micro-filtering films are implemented to obtain the optimal design parameters. First of all, in order to obtain a better pattern that fits the microfilters, two VOF models are established as shown in Figure 2. At different inlet velocities, a series of comparisons are conducted between micro-filtering films patterned with circular and slit-shaped pores. In these simulations, the contact angles are set as 180°, which means that the cell membrane is not attached to the films at all. As can be seen in Figure 3, the area strain of WBCs increases with the increase of the inlet velocity when cells transit through constrictions. At a inlet velocity of 0.175 mm/s, the WBCs only passed through the slit-shape patterned microfilter while at the inlet velocity of 0.625 mm/s, the WBCs only passed through the circular patterned one. Therefore, the slit-shaped microfilter has a higher sensitivity, as it can achieve the purpose of filtering out WBCs at a lower flow rate.

Generally, the area strain is composed of two parts: one part is derived from the impact that is caused by the inlet velocity and the other part is the basic area strain that is the minimal area strain needed for a larger cell transiting through smaller constrictions. The basic area strain can determined from Figure 3 as 5.1% and 15.3%, respectively. We found the report in the previous literature that area strains at lysis under low-rate dynamic loading were calculated to be 5%–6% [36,37,38], so the microfilter with 7 μm slit-shaped pores has advantages over the filter with circular pores. According to the aforementioned cellular injury criterion, we can infer that the circle patterned filtering film causes more harm to the passed WBCs. Additionally, note that the area strain that occurred in the circle patterned channel is much greater than the critical value of 5.5%, and therefore cell lysis may result. Additionally, the damaged cell membrane may stick to the surface of the microfilters so as to clog the pores.

**Figure 4:**
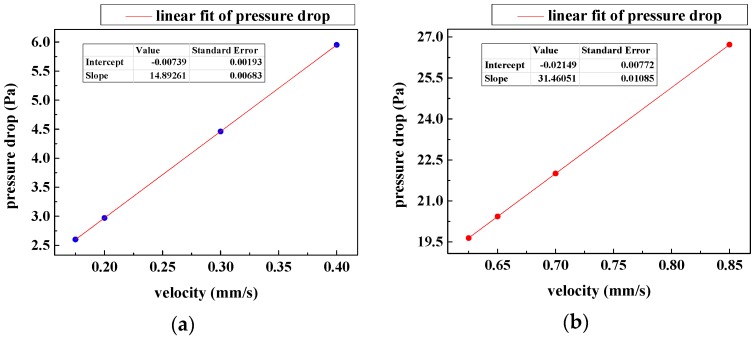
Pressure drop across the filtering films when WBCs are traversing at various inlet velocities: (**a**) pressure drop over the microfilter with slit-shaped pores; (**b**) pressure drop over the microfilter with circular pores.

Moreover, Figure 4 demonstrates the pressure drop across the filtering films versus the inlet velocity when WBCs pass through the microfilters. We observe that the pressure drop is proportional to the inlet velocity and the slope for circle patterned microfilters is greater. This implies that the cells traversing the circle patterned channel undergo larger forces, which may do more harm to the WBCs. In summary, the slit-shaped patterned microfilters are preferred because they cause less cellular damage.

#### 3.1.2. Cellular Damage of the Captured Circulating Tumor Cells (CTCs)

In order to achieve the filtering function, the CTCs should be captured. At the inlet velocity of 75 mm/s, the CTCs are only arrested by the slit-shaped patterned film and a higher velocity will lead to the 10 μm CTCs being filtered out. That is to say, all the CTCs including the ones with minimal sizes will be captured at inlet velocities less than 75 mm/s. When the CTCs enter small constrictions, the largest cell is the one with the largest damage. Thus, the captured CTCs with maximal sizes are analyzed here to ensure that all the damages that are caused are minimal. Under the same velocity conditions, the membrane area strain of 20 μm CTCs trapped in the microfilters are listed in Table 2. In the case of a contact angle of 90°, the area strain of 20 μm CTCs is up to 13% when trapped in the circular pores while it is 10% when trapped in the slit-shaped pores. However, if the contact angle is set as 180°, the area strains caused by the same velocity are much smaller since the affinity between the cells and filtering film has a considerable influence on the cellular deformation. The affinity can affect the form of contact between the cell and the film. If the filtering film is subjected to a surface treatment so as to exhibit strong hydrophilicity, the contact angle can be regarded as 180°. In this case, the cellular damage is minimal.

**Figure 5:**
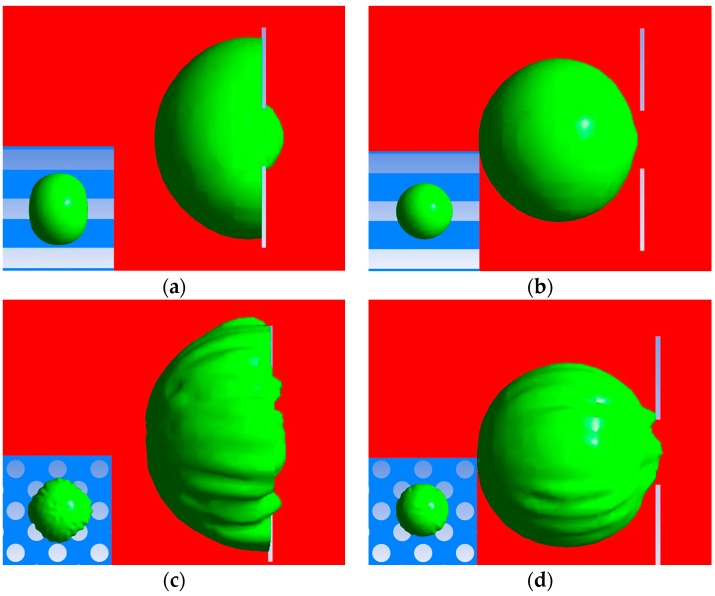
Circulating tumor cells (CTCs) captured in the microfilters at an inlet velocity of 75 mm/s: (**a**) CTCs captured in the slit-shaped pores with a contact angle of 90°; (**b**) CTCs captured in the slit-shaped pores with a contact angle of 180°; (**c**) CTCs captured in the circular pores with a contact angle of 90°; (**d**) CTCs captured in the circular pores with a contact angle of 180°.

The captured CTCs in the filtering films are illustrated in Figure 5. We observed a more turbulent flow of fluid through the circular pores, which caused wrinkles on the cytomembrane. Additionally, the pressure drops over the micro-filtering film with circular and slit-shaped pores are obtained as 2400 Pa and 1200 Pa, respectively. That is, in the case of the same flow rate and porosity, the force exerted on the cells by the circular pores is about twice as large as that by the slit-shaped pores. Taking the lower cellular injury requirement into account, the conclusion that the microfilters with slit-shaped pores are better is drawn due to their lower area strain and pressure drop. Since the slit-shaped patterned microfilters have significant advantages of lower cellular injury over their circular counterparts, the following research is all for slit-shaped microfilters with strong hydrophilicity, unless otherwise noted.

### 3.2. Characteristic Dimension Analysis

Since the slit-shaped filtering films are better, the next step is to carry out the study of the slit-shaped pattern more deeply. Firstly, we have to solve the problem of the selection of the characteristic dimension, i.e., the width of the rectangles, which restricts the passage of cells. Different characteristic dimensions have different effects on the filters. It will result in poor filtering efficiency and resolution if the characteristic dimension is too large; on the other hand, it will cause excessive cellular damage if the characteristic dimension is too small. An appropriate characteristic dimension is required to guarantee that the filtering films are capable of distinguishing CTCs as much as possible without damaging the cells.

**Figure 6:**
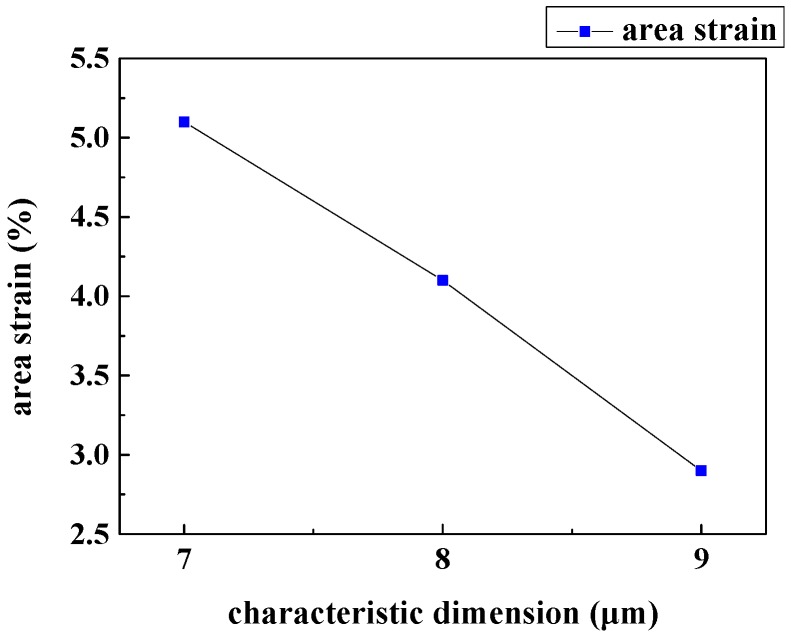
The effect of the characteristic dimension on the basic area strain of WBCs traversing slit-shape patterned microfilters.

The effect of the characteristic dimension on the basic membrane area strain of WBCs traversing slit-shape patterned microfilters is illustrated in Figure 6. This simulation result proves that choosing 7-µm as the characteristic dimension is reasonable; if the size is a bit larger, the resolution of the separation will be reduced, and if the size is smaller, the basic area strain will exceed 5%.

**Figure 7:**
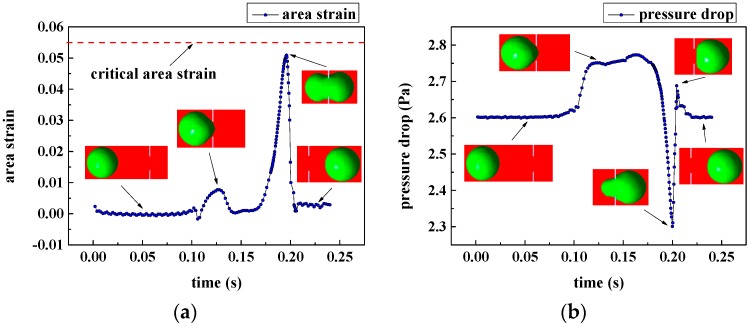
Area strain and pressure drop at the inlet velocity of 0.175 mm/s: (**a**) area strain of WBCs passing through the microfilter with slit-shaped pores; (**b**) pressure drop over the microfilter with slit-shaped pores.

In addition, the area strain and pressure drop between the filtering film with the 7 μm characteristic dimension are recorded in Figure 7.

### 3.3. Film Thickness Analysis

Next, the relationship between film thickness and pressure drop over the filtering film is investigated. As we all know, thickness is an important design parameter, and will directly guide the selection of fabrication materials. The pressure drop, *P*_total_, across the filtering film is governed by viscosity and boundary layer separation:

*P*_total_ = *P*_vis_ + *P*_bound_, (1)

**Figure 8:**
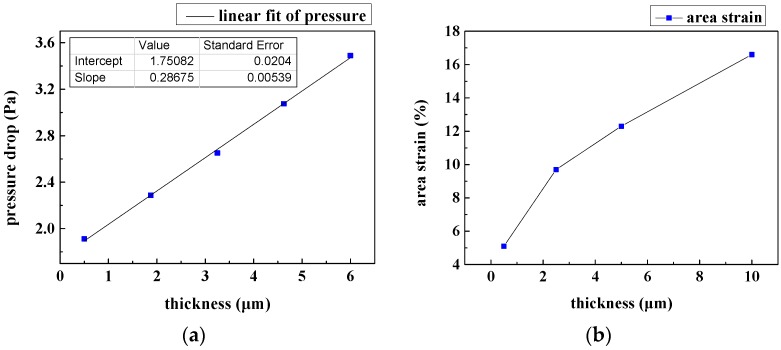
Effects of the thickness of slit-shaped patterned microfilters: (**a**) pressure drop between filtering films with different thickness; (**b**) area strain of WBCs passing through filtering films with different thicknesses.

Generally, the pressure drop caused by viscosity *P*_vis_ is proportional to the wetted perimeter, while the pressure drop caused by boundary layer separation *P*_bound_ is proportional to the trailing vortex intensity, which is inversely proportional to the characteristic dimension of the pores of the filtering film. Therefore, a thinner film will produce lower *P*_vis_, but *P*_bound_ is only affected by the film pattern. As can be seen in Figure 8a, there exists a minimal pressure drop of 1.75 Pa in the plot with decreasing film thickness while holding the inlet velocity at 0.1 mm/s. It is obvious that a lower pressure drop imposes a smaller force on cells and thus may do less damage. However, to attain lower cellular injury, we cannot merely rely on thinner film since a nonzero pressure drop exists even if the film thickness decreases to zero.

Moreover, the membrane area strain of 16 μm WBCs is shown in Figure 8b. We can see that the area strain increases sharply with increasing thickness. In this regard, the microfilters should be as thin as possible. Nevertheless, the filtering film cannot be considered as a rigid body any more if the thickness is too small. Eventually, a 0.5 μm thickness of the microfilters was selected as a compromise of fabrication techniques and stiffness of the film. Silicon nitride is an appropriate material to fabricate the microfilters. Due to the presence of internal stresses, even very thin silicon nitride films are stiff enough. The silicon nitride etched by fluorinated xenon can be as thin as 0.5 μm while still having sufficient strength.

### 3.4. Velocity Optimization

Finally, the inlet velocity that should be used in the separation experiments is discussed. In order to fulfill the separation function, the inlet velocity should fall within the range of the following two critical flow rates, at which the largest WBCs and the smallest CTCs can exactly pass right through the microfilters, respectively. The critical flow rates of the microfilter with the optimized thickness and pattern are obtained as shown in Table 3. Moreover, the filtered blood is to be re-filtered for further enrichment of CTCs or pharmacologically processed for follow-up analysis. This requires that the WBCs not be damaged in case the lysed WBCs clog the filtering film or impact the subsequent analysis. Therefore, the inlet velocity should be sufficiently small, so that the area strain is less than 5.5%. Then, the flow rate range is further limited between 0.175 mm/s and 0.375 mm/s, as illustrated in Figure 3a. If the flow rate is higher than 0.375 mm/s, the physical filtration will affect the cell viability, causing larger cellular injury. On the contrary, a flow rate less than 0.175 mm/s will lead to lower filtering efficiency since a certain subpopulation of WBCs may be trapped in the microfilters. Since a higher inlet velocity leads to higher throughput, the optimal velocity is finally determined as 0.375 mm/s.

## 4. Conclusions

To obtain the optimal design parameters for circulating tumor cell (CTC) microfilters, a series of simulations of the traversing processes of cells are implemented. In the simulations, we propound a preferable pattern for CTC filtration by comparison. That is, with the same characteristic dimension and porosity, the microfilters with slit-shaped pores are better than those with circular pores, and cause lower cellular injury. In addition, the optimal thickness of the CTC filtering films is determined as 0.5 μm. Furthermore, we found an optimal flow rate, i.e., 0.375 mm/s, to ensure that CTCs are captured with minimal cellular damage. Moreover, the impact of the contact angle is revealed. Namely, a 180° contact angle leads to minimal membrane area strain. Our study is valuable for the design of next generation microfilters with higher throughput, less cellular damage, and higher efficiency.

## Figures and Tables

**Table 1:** The model settings in the volume of fluid (VOF) simulation.

Parameters	Settings	Reference
CTC diameter	10 μm	-
WBC diameter	16 μm	-
Blood (main phase) viscosity	0.0035 Pa·s	[39]
Cell (secondary phase) viscosity	0.001 Pa·s	[40]
CTC surface tension	0.03 N/m	[41]
WBC surface tension	3 × 10^−5^ N/m	[20]

**Table 2:** The membrane area strain of circulating tumor cells (CTCs) trapped in the microfilters at an inlet velocity of 75 mm/s.

Pattern	90° Contact Angle	180° Contact Angle
Slit-shape	10%	around 0
Circle	13%	2.5%

**Table 3:** Critical flow rates of the microfilter with the optimal thickness and pattern.

Cell Type	Critical Velocity (mm/s)
CTC (10 μm)	75
WBC (16 μm)	0.175
